# Ultrahigh Storage Capacity of Alkali Metal Ions in Hexagonal Metal Borides with Orderly Multilayered Growth Mechanism

**DOI:** 10.3390/nano15120886

**Published:** 2025-06-08

**Authors:** Jiaxin Jiang, Hongyan Guo, Ning Lu

**Affiliations:** Department of Physics, Anhui Normal University, Wuhu 241000, China; jiangjiaxin@ahnu.edu.cn

**Keywords:** *h*-MBenes, secondary batteries, anode, DFT

## Abstract

The global energy shortage and the gradual depletion of lithium resources have become increasingly prominent. Improving the energy density of lithium-based secondary batteries and developing other high-performance alkali-metal secondary batteries have become the research focus. In this study, two-dimensional (2D) hexagonal metal borides (*h*-MBenes) are investigated as ordered alkali metal adsorption substrates for alkali-metal-based battery anode materials using density functional theory (DFT). Twelve thermodynamically stable *h*-MBenes are screened out from thirty-three structures, and their excellent stability and metallic electronic characteristics are confirmed. The ordered multilayered growth in alkali metal adsorption is found to depend on two factors: low lattice mismatching and dynamic matching of the work function. In particular, Mg/Al/V-based *h*-MBenes exhibit excellent lithium lattice matching (<3.35% mismatch), enabling layer-by-layer hexagonal (001) Li growth for ≥5 layers. They have ultrahigh lithium capacities (2170–3818 mAh·g^−1^), low migration barriers (0.01–0.05 eV), and low voltages (0.003–0.714 V). Mg/Y-based *h*-MBenes enable three Na layers’ adsorption with a capacity of 1717/605 mAh·g^−1^, and Al_2_B_2_ achieves a 472 mAh·g^−1^ potassium storage capacity, respectively. Due to the orderly multilayered growth mechanism, Mg/Al/V-based *h*-MBenes show great potential as high-safety and ultrahigh-capacity alkali-metal battery anode materials.

## 1. Introduction

With the global urgent demand for high-energy-density energy storage technologies, alkali metal anodes have demonstrated the highest known mass-specific capacities (including alkali metal mass) due to their host-free characteristics: lithium (3860 mAh·g^−1^), sodium (1166 mAh·g^−1^), and potassium (685 mAh·g^−1^) [1]. However, their uncontrollable dendrite growth, causing battery short circuits, remains the primary obstacle to large-scale applications. Compared with traditional liquid electrolyte systems, solid-state electrolytes can physically suppress alkali metal dendrite penetration through their high mechanical modulus while enabling the stable operation of anodes at ultralow potentials approaching 0 V through expanded electrochemical stability windows (0–5 V). This characteristic provides a foundation for lithium/sodium/potassium metal anode applications [2]. However, in solid-state systems, although dendrite issues are partially mitigated, repeated irregular metal deposition/stripping processes still induce volume changes, leading to electrode/electrolyte interface separation and ion transport channel collapse, resulting in significant capacity reduction [3,4].

To break through this bottleneck, designing the lattice morphology and electronic structure of the substrate material to induce layer-by-layer deposition of alkali metals has emerged as an effective solution combining a high energy density and safety. For example, Wei et al. successfully guided lithium metal to form planar deposition structures transitioning from hexagonal close-packed (*hcp*) interfacial layers to body-centered cubic (*bcc*) bulk phases on 2D Ti_3_C_2_T*_x_* MXene substrates, achieving ultra-stable cycling over a wide current range of 0.5–10 mA·cm^−2^ (capacity retention > 90% after 500 cycles at 1C rate) [5]. Xiao et al. developed a high-fluorine-flux solid electrolyte interface (F_3_-SEI) on silicon substrates through vapor fluorination [6]. The LiF nanocrystals and Si(CH_3_)_3_ organic components induced preferential lithium deposition along (110) crystal planes, realizing planar lithium layer growth with 99.2% compactness. These studies indicate that exploring substrate materials supporting layer-by-layer metal deposition represents a new direction to achieve both a high energy density and safety.

As a novel new 2D material family, metallic borides (MBenes) have recently shown unique application potential in energy storage [7]. Theoretical calculations reveal that *h*-MBenes with Mg/Al/V metal bases exhibit an outstanding performance in alkali metal ion battery anode applications, demonstrating theoretical lithium/sodium ion capacities of 342–968 mAh·g^−1^ and ultralow ion migration barriers (0.012–0.22 eV) [8,9,10]. These theoretical predictions provide crucial guidance for experimental research. Recently, Wang et al. combined a structural search with experimental verification to successfully synthesize hexagonal HfBO for the first time. This material maintains a reversible capacity of 420 mAh/g after 500 cycles at 0.1 A/g, with its accordion-like layered structure significantly enhancing ion transport kinetics [11]. Meanwhile, Xiong et al. obtained 2D MoB MBene through the exfoliation of MoAlB precursors, which demonstrates an excellent structural stability by retaining a Na-storage capacity of 138.6 mAh/g after 500 cycles [12]. However, the current research primarily focuses on liquid battery systems, requiring voltages no lower than the 0.1 V (vs. M^+^/M) metal deposition safety limit of traditional anodes [13]. Therefore, their potential as deposition substrates for alkali-metal-based anodes remains unexplored.

Compared with graphene [14] or other metal chalcogenides, *h*-MBenes have structural dynamic adjustability through diverse boron atom framework–metal atom pairings, offering rich surface characteristics and electronic state distributions. Combined with high-symmetry lattices and atomically flat surfaces, they form an ideal platform for studying and regulating controlled alkali metal deposition behaviors. In this study, 12 thermodynamically stable *h*-MBenes are screened out from 33 structures, and their multiscale characteristics as anode materials for alkali-metal-based batteries are systematically investigated through first-principles calculations. This reveals the physical mechanism of lattice mismatch and surface work function synergistically regulating layer-by-layer adsorption/growth, while demonstrating that Mg/Al/V-based *h*-MBenes significantly outperform traditional carbon-based materials in lithium metal adsorption capacity (>2000 mAh·g^−1^) and ultralow ion migration barriers (<50 meV). These findings provide new theoretical guidance for designing alkali-metal-based battery anodes with both high safety and high-rate performance.

## 2. Materials and Methods

### 2.1. Materials

In this study, we employ *h*-MBenes with a sandwich-like structure in the P6/mmm space group as the foundational model, where a central honeycomb boron (B) layer is stacked between two hexagonal close-packed (*hcp*) metal (M) layers. The metal component M encompasses 33 common metallic elements.

### 2.2. Calculation Details

This study is based on spin-polarized density functional theory (DFT) calculations conducted using the VASP software package, version 5.4. [15,16,17]. In all calculations, ion-electron interactions are described using projector augmented wave (PAW) pseudopotentials, with generalized gradient approximation (GGA-PBE) selected for the exchange-correlation functional. The DFT-D3 method is applied to correct the adsorption interactions of alkali metals [18], where alkali metal adsorption is simulated with neutral alkali atoms. The plane-wave basis cutoff energy is set to 500 eV, and structural optimizations are performed using the conjugate gradient (CG) algorithm, with energy and force convergence criteria of 10^−5^ eV and 0.01 eV/Å, respectively. A 20 Å pure vacuum layer is added along the c-axis to avoid periodic mirror interactions. Brillouin zone integration employs Γ-centered k-point meshes: k-point densities for unit cells, $\sqrt{3}$ × $\sqrt{3}$ × 1, and 2 × 2 × 1 supercells are set to 18 × 18 × 1, 12 × 12 × 1, and 9 × 9 × 1, respectively. For electronic structural calculations, these are further refined to 24 × 24 × 1, 18 × 18 × 1, and 12 × 12 × 1 to enhance accuracy. Lattice dynamic stability is verified through phonon spectra calculated using the PHONOPY code combined with density functional perturbation theory (DFPT) [19]. Thermodynamic stability is evaluated via ab initio molecular dynamics (AIMD) simulations on 5 × 5 × 1 supercells, with parameters including a constant temperature of 300 K, a 1.0 fs time step, and a total simulation duration of 5 ps.

The thermodynamic stabilities of *h*-MBenes are verified by computing formation energies (*E_f_*) according to

$$E_{f}=\left(E_{M_{2}B_{2}}-{2E}_{M}-2E_{B}\right)/4$$

Here, *E_M_* represents the energy per metal atom in the bulk metal phase (M-bulk), while *E*_B_ denotes the energy per boron atom in the β_12_ borophene monolayer.

The in-plane Young’s stiffness (*Y*) and Poisson’s ratio (ν) of the 2D system were calculated from the elastic constants using the following equations:

$$Y=\left(C_{11}C_{22}-{C_{12}}^{2}\right)/C_{11}$$

$$\nu =C_{12}/C_{11}$$

The stepwise layer-by-layer adsorption energy (*E_ads-step_*) was calculated using the following formula:

$$E_{ads-step}=\frac{E_{{AM}_{m}M_{2}B_{2}}-E_{{AM}_{n}M_{2}B_{2}}-\left(m-n\right)E_{AM}}{m-n}$$

where *m* and *n* represent the number of alkali metal ions adsorbed on the M_2_B_2_ surface, and *E*_AM_ denotes the energy of a single alkali metal atom in its corresponding body-centered cubic (bcc) phase.

Theoretical specific capacities (*C_M_*) for alkali metals were calculated by the following:

$$C_{M}=\frac{xzF}{M_{M_{2}B_{2}}}$$

where *x* represents the number of adsorbed alkali metal ions, *z* is the valence electron number of an alkali metal ion (*z* = 1), *F* denotes the Faraday constant (26,801 mAh·mol^−1^), and $M_{M_{2}B_{2}}$ indicates the molar mass of M_2_B_2_.

Adsorption energy (*E_ads_*) was calculated by the following:

$$E_{ads}=E_{AM@M_{2}B_{2}}-E_{M_{2}B_{2}}-E_{AM}$$

The formation energy of AM*_x_*M_2_B_2_ (*E_f-AM_*) was calculated by the following:

$$E_{f}=\frac{E_{{{AM}_{x}M}_{2}B_{2}}-E_{M_{2}B_{2}}-xE_{AM}}{x+1}$$

The open-circuit voltage (*Voc*) was obtained by the following:

$$Voc=-\frac{E_{{AM}_{m}M_{2}B_{2}}-E_{{AM}_{n}M_{2}B_{2}}-\left(m-n\right)E_{AM}}{\left(m-n\right)e}$$

The lattice mismatch was obtained from the following:

$$\varepsilon =\frac{L_{{{AM}_{x}M}_{2}B_{2}}-L_{AM-hcp}}{L_{AM-hcp}}\times 100\%$$

where $L_{{{AM}_{x}M}_{2}B_{2}}$ and $L_{AM-hcp}$ represent the lattice constants of the system after adsorbing *x* alkali metal ions and the lattice constant of the AM in its *hcp* phase, respectively.

## 3. Results and Discussion

### 3.1. Structure and Stability of h-MBenes

**Figure 1:**
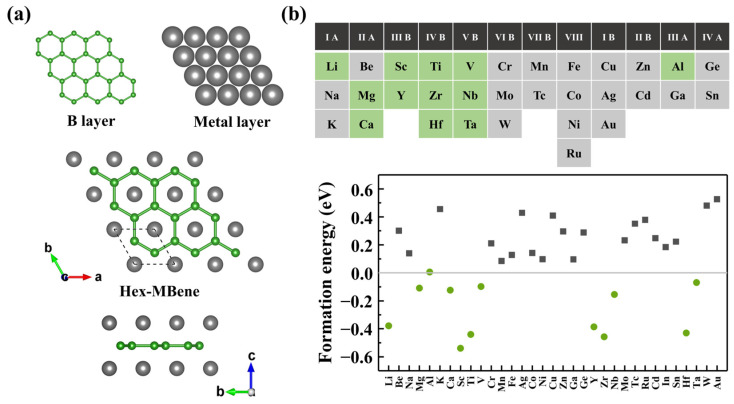
(**a**) The crystal structure of *h*-MBenes (M_2_B_2_). (**b**) The formation energies for 33 types of *h*-MBenes. Here, the green square elements represent candidate metal elements with negative formation energies. In the formation energy plot, black squares denote positive formation energies, while green circles indicate negative ones.

Two-dimensional *h*-MBenes exhibit a characteristic sandwich-like structure with *P6/mmm* space group symmetry, formed by stacking a central honeycomb boron (B) layer between two hexagonal close-packed metal (M) layers, resulting in an M-B-M trilayer configuration. The unit cell has a stoichiometry of M_2_B_2_ (containing two M atoms and two B atoms), as shown in Figure 1a. In this work, 33 representative metallic elements are selected to construct various *h*-MBene configurations. Their thermodynamic stabilities are verified by calculating formation energies (*E_f_*). The calculated results reveal that M_2_B_2_ systems with M = Li, Mg, Ca, Sc, Ti, V, Nb, Zr, Y, Hf, and Ta exhibit negative *E_f_* values (−0.098 to −0.539 eV/atom), indicating spontaneous thermodynamic formation (Figure 1b). Notably, Al_2_B_2_ shows a slightly positive *E_f_* value of +0.005 eV/atom, marginally above the thermodynamic stability limit. Consequently, 12 *h*-MBenes are selected for a systematic investigation, including 9 previously reported systems (Mg-, Al-, Ca-, Sc-, Ti-, V-, Nb-, Zr-, and Y-based *h*-MBenes) [9,10,13,20,21,22], which are theoretically predicted as promising alkali metal ion battery anode materials. The Li-, Hf-, and Zr-based *h*-MBenes are first proposed in this study, expanding the theoretical candidate for 2D metallic borides. The structural information of these 12 M_2_B_2_ systems is statistically summarized in Table S1.

To validate structural stability, ab initio molecular dynamics (AIMD) simulations at 300 K for 5 ps confirm there are no structural distortions or disordering (Figure S1), demonstrating excellent thermal stability in ambient conditions. Further lattice phonon dispersion calculations (Figure S2) show no imaginary frequencies across the entire Brillouin zone (Γ-M-K-Γ), verifying the strong lattice dynamic stability.

### 3.2. Mechanical and Electronic Properties of h-MBenes

The in-plane mechanical properties of *h*-MBene monolayers are characterized by calculating their elastic constants. All systems satisfy the Born–Huang mechanical stability criteria (*C*_11_*C*_22_ − *C*_12_^2^ > 0 and *C*_66_> 0) [23], confirming their good mechanical stability. Owing to high in-plane symmetry, these materials exhibit isotropic mechanical behavior. However, significant variations in mechanical strength are observed across different *h*-MBene structures. The elastic constants *C*_11_ = *C*_22_ range from 113.8 to 246.7 N/m, *C*_12_ = *C*_21_ from −0.8 to 82.4 N/m, and *C*_66_ from 48.5 to 112.9 N/m. The in-plane Young’s stiffness (Y) and Poisson’s ratio (ν) of the 2D system are calculated. The calculated results show the in-plane *Y* ranges of 111.3–239.9 N/m, with ν ranging from −0.005 to 0.393. Notably, Al-, Sc-, Ti-, V-, Zr-, Y-, Hf-, and Ta-based *h*-MBenes exhibit *Y* exceeding 200 N/m, indicating the superior mechanical strength. In contrast, Ca_2_B_2_ shows the lowest *Y* of 111.3 N/m, attributed to its largest lattice constant and weakened covalent interactions due to elongated B-B bonds. Among Poisson’s ratio, Nb_2_B_2_ and Ta_2_B_2_ display ν values above 0.3, while Mg-, Sc-, Ti-, and Y-based systems exhibit ν values below 0.1. Remarkably, Li_2_B_2_ demonstrates an ultralow ν value of −0.005, classifying it as a rare near-zero Poisson’s ratio material [24]. These diverse mechanical properties highlight the potential of *h*-MBenes for applications in electrode materials and flexible electronics.

**Figure 2:**
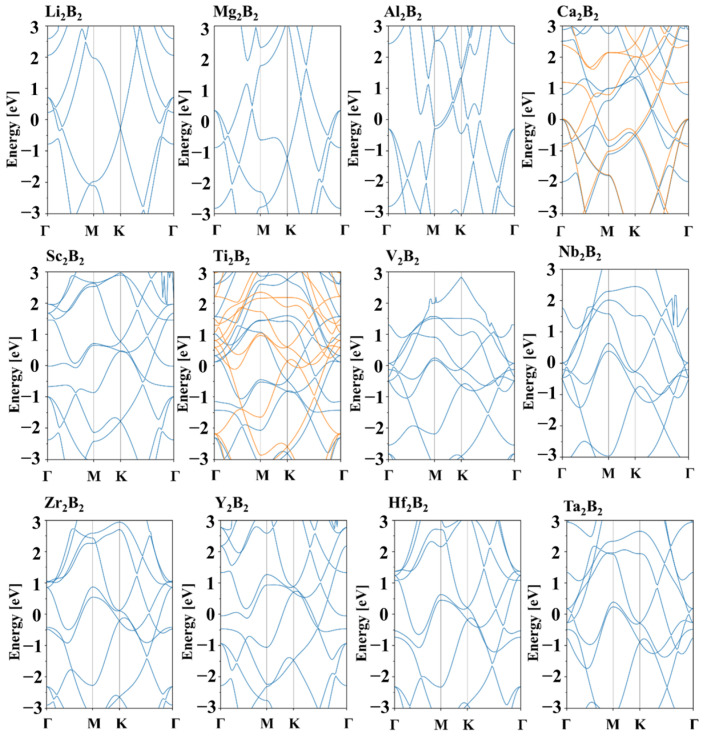
The band structures of 12 *h*-MBenes calculated based on the PBE functional, with the Fermi level set at 0 eV. Here, for Ca_2_B_2_ and Ti_2_B_2_, the blue and orange lines represent the spin-up and spin-down electronic bands, respectively.

Electronic structural calculations based on the PBE functional reveal metallic characteristics for all *h*-MBenes, with continuous electronic states at the Fermi level (Figure 2). Among these, Ca_2_B_2_ and Ti_2_B_2_ exhibit unique magnetic behavior. Projected partial wave density of states (PDOS) analysis (Figure S3) indicates that the valence bands of *h*-MBenes are dominated by boron 2*p* orbitals, while conduction bands arise from the outer orbitals of the metal atoms. The excellent electrical conductivity of *h*-MBenes highlights their potential as a high-performance anode material for alkali-metal-based batteries.

### 3.3. The Storage Capacity and Migration of Alkali Metal on h-MBenes

In electrochemical energy storage systems using metal cations as charge carriers, while low anode voltages can enhance full-cell voltage output, traditional liquid electrolyte systems typically require anode potentials no lower than 0.1 V (vs. M^+^/M) to avoid dendrite formation. Advances in all-solid-state battery technology resolve this conflict: solid-state electrolytes physically suppress dendrite penetration via high mechanical strength and enable stable anode operation at an ultralow voltage near 0 V. This is not only convenient for the practical application of alkali metal anodes but also can achieve an ultrahigh specific capacity. Notably, encouraging ordered layer-by-layer adsorption of alkali metals via specific substrates is an effective strategy to enhance anode performance. Thus, developing alkali metal anodes based on advanced substrate materials is critical for advancing high-energy-density battery technologies.

**Figure 3:**
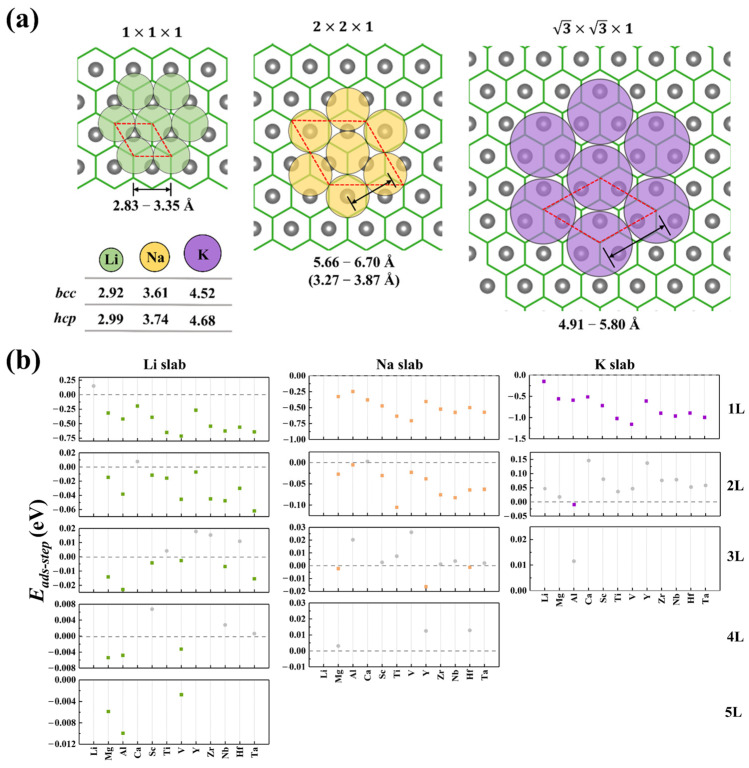
(**a**) Schematic illustration of the hexagonal close-packed (*hcp*) adsorption configuration of alkali metals (AMs) on the *h*-MBene surface. The bottom-left inset shows the minimum AM-AM bond length in the bulk (*bcc/hcp*) phase of alkali metals. The red dashed box indicates the minimal supercell lattice under this stacking mode, with the data below each structure representing the corresponding lattice parameter range of different *h*-MBene materials. (**b**) Variation in the stepwise adsorption energy (*E_ads-step_*) with layer number during the adsorption of alkali metal layers on different *h*-MBene surfaces. Here, the color dot (green: Li; orange: Na; purple: K) represent negative *E_ads-step_* values, while the gray ones indicate positive values.

The atomically flat surfaces of *h*-MBenes allow alkali metals to deposit layer by layer along specific crystallographic planes, which was called an “orderly multilayered growth mechanism” in our previous work [25]. This controlled growth mode suppresses dendrite formation by eliminating localized charge accumulation and stress concentration. A key factor enabling controlled growth is lattice matching between the substrate and alkali metal crystal planes. Excessive lattice mismatch (>critical value) induces interfacial stress accumulation, raising surface energy and destabilizing layer-by-layer growth, leading to dendrite formation. As shown in Figure 3a, alkali metals (AMs) preferentially adsorb/grow on *h*-MBene surfaces via the *hcp*-phase (001) plane (see details in Figure S4), a mechanism similar to the experimentally observed *hcp*-Li layer growth on Ti_3_C_2_T_x_ substrates [5]. Systematic analysis of *h*-MBene lattice parameters reveals that their 1 × 1 × 1, 2 × 2 × 1, and $\sqrt{3}$ × $\sqrt{3}$ × 1 supercell constants have lengths of 2.83–3.35 Å, 5.66–6.70 Å, and 4.91–5.80 Å, respectively. The *hcp*-phase (001) plane lattice dimensions for Li, Na, and K are 2.99 Å (Li unit cell), 6.48 Å (Na $\sqrt{3}$ supercell), and 4.52 Å (K unit cell). The *h*-MBene supercells exhibit excellent lattice matching with Li and Na, except K. Thus, 1 × 1 × 1, 2 × 2 × 1, and $\sqrt{3}$ × $\sqrt{3}$ × 1 supercells are used to study Li, Na, and K layer growth, respectively.

The maximum adsorption layer capacity of various *h*-MBenes for alkali metals is determined by calculating the stepwise layer-by-layer adsorption energy (*E_ads-step_*). When *E_ads-step_* is negative, it indicates effective adsorption at that layer, while a positive value signifies the termination of further layer adsorption. As shown in Figure 3b, *h*-MBene substrates exhibit distinct layer-by-layer adsorption capacities for different alkali metals. For lithium, Mg-, Al-, and V-based *h*-MBenes demonstrate excellent layer growth potential. The calculated *E_ads-step_* remains negative (−10 to −2 meV/atom) even at the fifth layer (Li_10_M_2_B_2_ stoichiometry), with E_step_ stabilizing as layers increase. In contrast, sodium and potassium exhibit limited layer growth. Mg-, Y-, and Hf-based *h*-MBenes achieve three sodium layers (Na_4_._5_M_2_B_2_), while the fourth layer of adsorption shows a positive *E_ads-step_* (+3/+13/+13 meV/atom). For potassium, only Al_2_B_2_ supports two stable K layers (K_1_._33_Al_2_B_2_), with the third layer of adsorption showing a positive *E_ads-step_* (+12 meV/atom). Note that Li_2_B_2_ is excluded due to the unstable lithium adsorption in Li and Na systems. Detailed *E_ads-step_* data are provided in Tables S2–S4. Based on the saturated adsorption stoichiometry of each system, Mg-, Al-, and V-based *h*-MBenes achieve ultrahigh theoretical specific capacity ($C_{M}$) values of 3818, 3544, and 2170 mAh·g^−1^ for Li, respectively. Mg-, Y-, and Hf-based systems reach 1717, 605, and 318 mAh·g^−1^ for sodium, respectively. And Al_2_B_2_ achieves 472 mAh·g^−1^ for potassium (Figure S5). These values are higher than graphite’s capacities for Li (372 mAh·g^−1^) and K (279 mAh·g^−1^) [26]. For more comparisons, for Li storage of Mg- and Al-based M_2_B_2_, their capacities are higher than those of borophene (1860 mAh·g^−1^) [27] and B_7_P_2_ (3117 mAh·g^−1^) [28]. For Na storage of Mg_2_B_2_, the capacity is much higher than that of phosphorene (865 mAh·g^−1^) [29]. For K storage of Al_2_B_2_, the capacity is higher than that of graphite (279 mAh·g^−1^) [26] but much lower than that of B_3_P (1473 mAh·g^−1^) [30], highlighting their potential for high-capacity energy storage applications.

**Figure 4:**
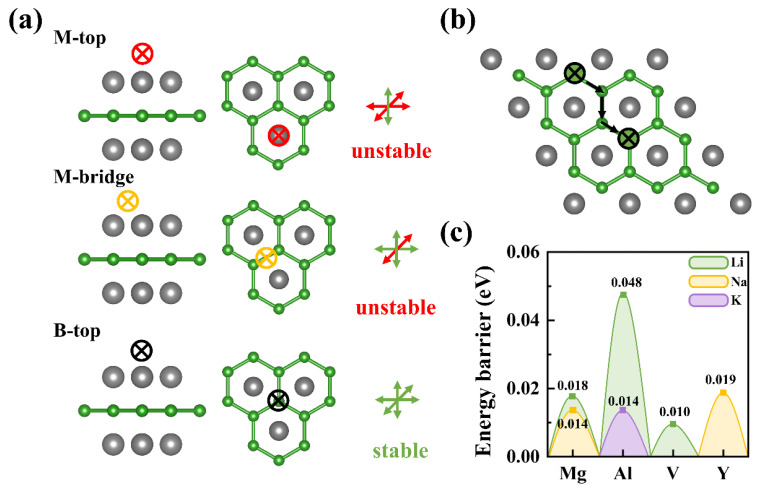
(**a**) The adsorption sites and stability analysis for alkali metal atoms on a *h*-MBene monolayer surface. Here, the gray and green balls represent M and B atoms, respectively, while the colored circular × symbols denote adsorption sites. (**b**) Migration pathways of alkali metal ions on the *h*-MBene monolayer surface, and (**c**) the corresponding migration energy barriers.

Rapid migration of alkali metal ions on substrate surfaces is critical for achieving stable layer-by-layer growth and enhancing battery rate performance. Based on energy storage capacity variations among *h*-MBene systems, the best candidates of Li@Al/Mg/V-, Na@Mg/Y-, and K@Al-based *h*-MBenes are selected to construct 4 × 4 × 1 supercell models for studying single alkali metal atoms’ adsorption and migration. There is high symmetry of *h*-MBenes’ resulting alkali metals with a single stable adsorption site, the B-top site, where the alkali metal atom forms an equilateral three-coordinated structure with three adjacent metal atoms (M) (Figure 4a). In contrast, M-top and M-bridge sites offer only one and two coordinated environments, respectively, and cannot offering stable adsorption.

Structural optimization revealed Li-M bond lengths of 3.02/2.74/2.89 Å on Mg/Al/V-based *h*-MBenes, Na-M bond lengths of 3.32/3.70 Å on Mg/Y-based systems, and a K-Al bond length of 3.46 Å on Al_2_B_2_. The calculated results show that V_2_B_2_ exhibits the lowest adsorption energy (*E_ads_*) for Li (−0.985 eV), Y_2_B_2_ for Na (−0.520 eV), and Al_2_B_2_ for K (−1.111 eV), indicating significant variations in binding strength across these systems (Table 1). The charge density difference (Figure S6) confirmed the electron transfer from alkali metals to the M_2_B_2_ surface, forming stable chemical bonds. Bader charge analysis showed that the electron loss of most alkali metal atoms was greater than 0.83 e^−^ (Table 1), corresponding to the typical +1 valence state of alkali metals, except for Na@Y_2_B_2_ (0.579 e^−^). This stable adsorption provides a foundation for ion migration.

Migration energy barriers (*E_b_*) (Figure 4b,c) reveal the ultralow barriers for alkali metal ions moving along the B-B framework, Li (10–48 meV), Na (2–19 meV), and K (11 meV), and more data on *E_b_* is summarized in Table 1. These values are significantly lower than those of graphite (Li: 400 meV, K: 190 meV) [31] and other 2D carbon-based anodes like X-graphene (Li: 490 meV, Na: 330 meV, K: 180 meV) [32] and Hd-graphene (Li: 210 meV, Na: 140 meV, K: 90 meV) [33]. The combination of strong adsorption and rapid ion transport in *h*-MBenes ensures stable deposition/stripping behavior under high-rate conditions.

### 3.4. Open-Circuit Voltage and Thermal Stability of h-MBene Anodes

**Figure 5:**
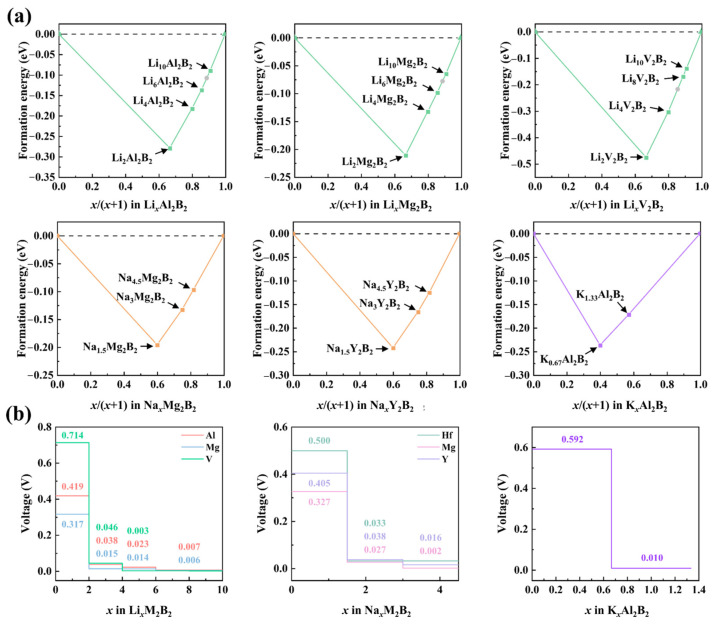
(**a**) Formation energy convex hull for various *h*-MBene materials during alkali metal adsorption at different concentrations, and (**b**) the average open-circuit voltage of anodes based on thermodynamically stable structures.

To evaluate the electrochemical performance of *h*-MBenes as anodes of alkali-metal-based batteries, the formation energy (*E_f-AM_*) of the convex hull of AM_x_M_2_B_2_ (AM = Li/Na/K) is calculated to identify thermodynamically stable intermediates and corresponding voltage plateaus during layer adsorption. As shown in Figure 5a, most intermediate phases in Li@Al/Mg/V-, Na@Mg/Y-, and K@Al-based M_2_B_2_ lie on the convex hull except for Li_2_Al_2_B2, Li_8_Mg_2_B_2_, and Li_6_V_6_B_6_. Based on stable intermediates, the open-circuit voltage (*Voc*) results show that each system exhibits 2–4 voltage plateaus (Figure 5b) during layer-by-layer growth. Discharge voltage ranges are 0.317–0.006 V (Li@Mg), 0.419–0.007 V (Li@Al), 0.714–0.003 V (Li@V), 0.327–0.002 V (Na@Mg), 0.405–0.016 V (Na@Y), and 0.592–0.010 V (K@Al). Notably, all systems approach 0 V (vs. AM^+^/AM) at the final discharge stage, enabling a stable alkali metal process near the thermodynamic limit for high-energy-density solid-state batteries.

Considering lithium’s known alloy-forming characteristics with Al and Mg, the ternary-phase diagrams [34] of Li_2_Mg_2_B_2_/Li_2_Al_2_B_2_ are calculated to investigate the potential formation of Li-Mg/Al alloys during lithiation (calculated details are shown in SI). As shown in Figure S7, the results reveal that within the allowable chemical potential range, Li_2_Mg_2_B_2_ exhibits a distinct stable-phase region, whereas the stable-phase region of Li_2_Al_2_B_2_ is negligible. This indicates that Li_2_Mg_2_B_2_ is thermodynamically more favorable for formation during lithiation, while Li_2_Al_2_B_2_ tends to form a mixed phase of Li-Al alloy and Al_2_B_2_. However, it should be noted that calculations based on bulk alloy energy references for 2D systems have limited reference value. Further AIMD simulations at 300 K confirm that both Mg_2_B_2_ and Al_2_B_2_ maintain excellent thermal stability at Li_10_M_2_B_2_ (M = Al/Mg), showing no alloying reactions between Li and Al/Mg layers. This confirms that Mg_2_B_2_ and Al_2_B_2_ can preserve their structure under limited Li layer adsorption conditions. Additionally, AIMD simulations at 300 K are performed for fully adsorbed Li@V-, Na@Mg/Y-, and K@Al-based *h*-MBenes, confirming their structural thermal stability (Figure S8). All systems maintained their lattice frameworks, with alkali metal layers stably attached, ensuring a high performance under working conditions.

In addition to the inherent stability of the anode, the stability of the electrode–solid electrolyte interface is crucial for the practical application of all-solid-state batteries. Currently, some effective strategies have been developed to enhance the chemical and mechanical stability of electrode–electrolyte interfaces. For instance, recent studies have demonstrated that constructing artificial Solid Electrolyte Interphase (SEI) buffer layers with 3D porous channels can effectively homogenize the Mg^2+^ distribution, enabling uniform Mg metal deposition and achieving high-performance Mg metal anodes [35]. Moreover, compared to pure metal anodes, h-MBene substrates can provide a platform for ordered multilayer growth of alkali metals, mitigating dendrite formation caused by disordered deposition. Additionally, employing 2D materials as growth substrates can effectively suppress mechanical degradation induced by volume expansion.

### 3.5. Layer-by-Layer Growth Behavior of Alkali Metals on h-MBene Surfaces

**Figure 6:**
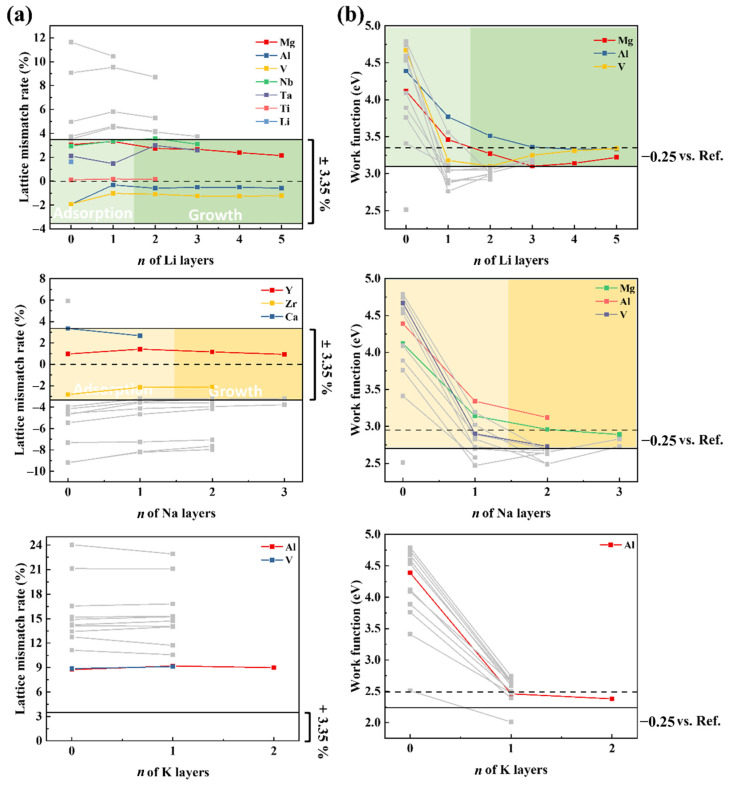
(**a**) The change in lattice mismatch ratio (%) and (**b**) surface work function with increasing layers of adsorbed Li, Na, and K on different *h*-MBene surfaces. Here, the colored lines represent data that meet the criteria, while the gray ones indicate non-conforming data. For the K-layer adsorption system, the colored lines here denote the data closest to the criteria.

Finally, to understand the differences in the orderly multilayered growth of alkali metals (AMs) on various *h*-MBene surfaces, the lattice mismatch rates (*ε*) and surface work functions (WFs) during the layer-by-layer adsorption process for each system are analyzed, as shown in Figure 6, and the corresponding data are summarized in Tables S5–S10. The adsorption and growth process of AMs on 2D substrates involves two stages: adsorption (first-layer AM bonding with substrate) and growth (subsequent layer-by-layer growth). During the adsorption stage, AM layers act as electron givers, transferring electrons to the substrate to form stable chemical bonds. Thus, the substrate WF must exceed that of the adsorbed layer. Taking Li_2_B_2_ as an example, its WF (2.51 eV) is lower than those of *hcp*-phase Li/Na (001) surfaces (Li: 3.35 eV, Na: 2.95 eV), hindering electron transfer from Li/Na layers to the substrate and causing adsorption failure of the first layer. In contrast, the WF of Li_2_B_2_ (2.51 eV) is slightly higher than that of *hcp*-K (2.49 eV), which helps the first K-layer adsorption to succeed. Additionally, artificial modulation of the WF can effectively regulate the adsorption of Li layers on Li_2_B_2_ and *hcp*-Li bilayers, as detailed in Figure S9. Except Li_2_B_2_, other *h*-MBene systems exhibit higher WFs than corresponding AM layers while maintaining sufficient surface-delocalized electrons (Figures S10 and S11) and satisfy electron transfer requirements for stable first-layer adsorption. Bader charge analysis (Table S11) and the charge density difference (taking Mg_2_B_2_ as an example, Figure S12) provide further evidence, demonstrating a consistent charge transfer from the first alkali metal adsorption layer to the substrate.

During the growth stage, continued electron transfer to interlayer regions to form metal-like interlayer interactions is crucial for stable multilayer growth. The WF of the system will be reduced after the adsorption of the first layer. When the WF approaches the bulk AM reference value, it balances charge transfer between the substrate and subsequent layers, similar to the process of spontaneous AM growth. However, much WF reduction to significantly below the bulk alkali metal reference badly affects interlayer electron sharing between subsequent layers and the substrate, hindering continued growth. Successful multilayer growth thus requires a system WF dynamically matched to bulk AM references throughout adsorption/growth. Besides the influence of the WF, lattice matching is also critical, and similar atomic arrangements (e.g., hcp/bcc-specific planes) and low lattice mismatch (ε) minimize strain energy from atomic misalignment. For instance, Mg-/Al-/V-based *h*-MBenes capable of adsorbing five Li layers maintain WF deviations < 0.25 eV relative to *hcp*-Li (3.35 eV) with the *ε* in the range of −0.31%~3.32%. Although Ti_2_B_2_ shows a minimal *ε* (0.12%~0.19%), its WF is 0.35–0.45 eV below the reference value, limiting Li adsorption to two layers. For Na growth on Mg_2_B_2_, it has minimal WF deviations (0.06~0.19 eV relative to hcp-Na (2.95 eV)) but with a high ε (−4.59%~−3.78%), limiting adsorption to only three layers. For the growth of K, due to the larger atomic radius of K, all *h*-MBene systems exhibit a high lattice mismatch exceeding 8% with K, limiting growth to a maximum of two layers. Additionally, the larger atomic radius of K also results in a significantly lower number of K atoms compared to Li and Na adsorbed on the same cell, leading to the lowest specific storage capacity. In summary, the requirements of orderly multilayered growth of AM on *h*-MBenes are different in the two stages. In the adsorption stage, it demands a higher substrate WF than the AM layer, and the growth requires two criteria: 1. dynamic matching of the WF to bulk AM references (e.g., *h*-MBene/AM system deviations ≤ 0.25 eV); 2. low lattice mismatch (e.g., *h*-MBenes/AM systems ≤ ±3.35%).

**Figure 7:**
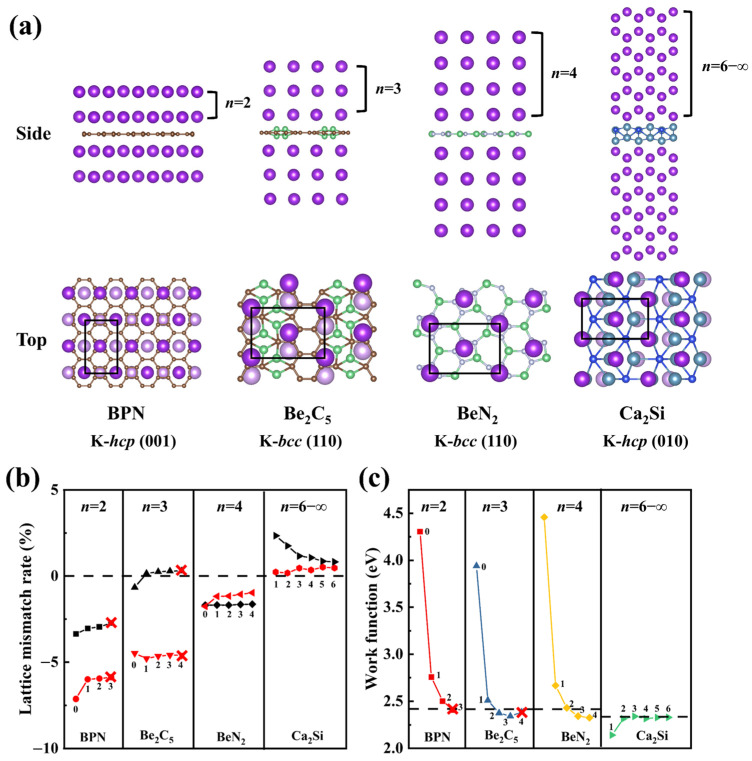
(**a**) Side views of four selected 2D systems after multilayer K adsorption, and top views of their structures with monolayer K adsorption. The black boxes indicate reference lattices for comparison. The change in lattice mismatch rate (**b**) and work function (**c**) of four selected 2D systems during multilayer K adsorption. In (**b**), the black and red dotted lines represent the lattice mismatch rate data along the *a*- and *b*-axis, respectively. Data for BPN and Be_2_C_5_ are plotted up to the 3rd and 4th layers (red × marks indicate adsorption failure), respectively. BeN_2_ data extend to the effective 4th layer. For Ca_2_Si, where first-layer adsorption induces phase transformation, data are collected from the 1st to 6th layers.

To verify the universality of the proposed theoretical criteria across diverse systems, four representative 2D materials with distinct potassium multilayer adsorption behaviors are selected for validation, a biphenylene (BPN) monolayer [36], 2D-Be_2_C_5_ [37], α-2D-BeN_2_ [38], and 2D-Ca_2_Si [25]. As shown in Figure 7a, these materials exhibit significant differences in multilayer potassium adsorption capacity and behavior. The BPN monolayer supports only bilayer adsorption of *hcp*-phase (001) potassium, while 2D-Be_2_C_5_ and α-2D-BeN_2_ achieve three and over four layers of *bcc*-phase (110) potassium, respectively. In contrast, 2D-Ca_2_Si demonstrates continuous *hcp*-phase (010) potassium growth (up to six layers in this study), consistent with reported results [25,36,37,38].

Lattice mismatch and surface WF evolution during multilayer adsorption are shown in Figure 7b,c. BPN and 2D-Be_2_C_5_ with limited K-layer adsorption maintain lattice mismatches exceeding 5% along the *b*-axis relative to their corresponding potassium crystal planes. In contrast, α-2D-BeN_2_ and 2D-Ca_2_Si, which enable multilayer adsorption, achieve excellent lattice mismatch (≤2%). Notably, the *a*-axis mismatch of 2D-Ca_2_Si converges to <0.5% as layers increase. WF evolution follows, with the criteria of all systems gradually converging toward the WF of their respective potassium crystal planes. Remarkably, 2D-Ca_2_Si achieves near-perfect WF matching (3 meV deviation from *hcp*-phase (010) reference) after the second layer of adsorption, maintaining stable fluctuations.

These results strongly confirm the theoretical criteria. 2D-Ca_2_Si enables sustained potassium epitaxial growth through perfect WF matching and dynamically converging lattice parameters, while α-2D-BeN_2_ achieves stable adsorption of at least four layers via both criteria. BPN and 2D-Be_2_C_5_ are forced by mismatches >5%. Furthermore, the tested systems cover three potassium crystal planes (*hcp*-001/010, *bcc*-110) and follow the same principles, confirming the universality of the criteria across diverse crystal structures. Whether these criteria extend to multidimensional substrates and diverse metal growth systems needs further exploration.

## 4. Conclusions

In conclusion, the structural stability, mechanical/electronic properties of two-dimensional hexagonal metallic borides (*h*-MBenes), and their performance as alkali-metal-based battery anodes for lithium/sodium/potassium storage are systematically investigated. Twelve thermodynamically stable *h*-MBene structures (M = Li, Mg, Al, Ca, Sc, Ti, V, Nb, Zr, Y, Hf, and Ta) are selected through formation energy calculations. Phonon spectra and AIMD simulations confirmed the lattice dynamic stability and thermal stability of all systems at room temperature. Among them, seven *h*-MBenes exhibit excellent mechanical strength, with Young’s stiffness exceeding 200 N/m, while Li_2_B_2_ demonstrates a unique near-zero Poisson’s ratio (−0.005). Electronic structural analysis revealed the metallic conductivity across all *h*-MBenes. Al/Mg/V-based *h*-MBenes demonstrate outstanding lithium-layer growth capabilities, achieving theoretical specific capacities of 2170–3818 mAh·g^−1^ with negative stepwise adsorption energies even beyond five layers. And the migration barriers of Li (10–48 meV) are significantly lower than those of traditional graphite anodes. For sodium and potassium, Mg/Y-based and Al-based *h*-MBenes achieve three and two adsorption layers, respectively, with capacities of 1717–605 mAh·g^−1^ (Na) and 472 mAh·g^−1^ (K). Convex hull analysis of formation energy identified stable voltage plateaus during charge/discharge: Li (0.006–0.714 V), Na (0.002–0.405 V), and K (0.010–0.592 V), showing significant potential of *h*-MBenes as high-safety, high-energy-density anodes for alkali-metal-based batteries. Finally, the criteria for ordered multilayer growth of alkali metals on *h*-MBenes are proposed: the WF of the substrate should higher than that of the AM reference in the adsorption stage; in the layer-by-layer growth stage, dynamic matching of the WF to bulk references (e.g., *h*-MBene/AM system deviations ≤ 0.25 eV), and low lattice mismatch (e.g., *h*-MBenes/AM systems ≤ ±3.35%) are needed. These criteria have been further confirmed in other 2D materials, providing a theoretical foundation for developing low-dimensional functional materials that suppress dendrite growth and enable ordered alkali metal adsorption/deposition with the orderly multilayered growth mechanism.

## Figures and Tables

**Table 1:** Adsorption energy (*E_ads_*), Bader charge transfer (Δ*e*), alkali metal–metal bond length (*L*_AM-M_), and migration energy barrier (*E_b_*) for single alkali metal (AM) ion adsorption on various *h*-MBene surfaces.

AM@M_2_B_2_	*E_ads_* (eV)	Δ*e* (e)	*L*_AM-M_ (Å)	*E_b_* (eV)
Li@Mg_2_B_2_	−0.215	−0.84	3.02	0.018
Li@Al_2_B_2_	−0.488	−0.86	2.74	0.048
Li@V_2_B_2_	−0.985	−0.85	2.89	0.010
Na@Mg_2_B_2_	−0.488	−0.70	3.32	0.014
Na@Y_2_B_2_	−0.520	−0.58	3.70	0.019
K@Al_2_B_2_	−1.111	−0.84	3.46	0.014

## Data Availability

The data that support the findings of this study are available from the corresponding author upon reasonable request.

## Supplementary Materials

The following supporting information can be downloaded at https://www.mdpi.com/article/10.3390/nano15120886/s1, Table S1. The lattice constant, formation energy, elastic constants, Young’s stiffness, and Poisson’s ratio of selected 12 *h*-MBenes. Table S2. The *E_ads-step_* of Li*_x_*M_2_B_2_. Table S3. The *E_ads-step_* of Na*_x_*M_2_B_2_. Table S4. The *E_ads-step_* of K*_x_*M_2_B_2_. Table S5. The lattice mismatch rate of Li*_x_*M_2_B_2_. Table S6. The lattice mismatch rate of Na*_x_*M_2_B_2_. Table S7. The lattice mismatch rate of K*_x_*M_2_B_2_. Table S8. The work function of Li*_x_*M_2_B_2_. Table S9. The work function of Na*_x_*M_2_B_2_. Table S10. The work function of K*_x_*M_2_B_2_. Table S11. The Bader charge analysis (*Δe*) per alkali metal atom in the first adsorbed alkali metal layer, where a negative value indicates electron loss. Figure S1. The AIMD simulations of selected 12 *h*-MBenes (T = 300 K, time = 5 ps). Figure S2. The phonon spectra of selected 12 *h*-MBenes. Figure S3. The PDOS of selected 12 *h*-MBenes under PBE level. Figure S4. The adsorption configurations and energy differences of each Li layer on Al_2_B_2_ and Mg_2_B_2_ substrates, with the most stable structure in each model as the energy reference. Figure S5. Theoretical specific capacity (mAh·g^−1^) comparison of various *h*-MBenes for alkali metals (Li/Na/K). Figure S6. Charge density difference of various alkali metal ions (Li/Na/K) adsorbed on different *h*-MBenes surfaces. Figure S7. Thermodynamic phase diagrams of lithiated (a) Li_2_Mg_2_B_2_ and (b) Li_2_Al_2_B_2_ structures. Figure S8. AIMD simulations of the saturated adsorption systems for performance-optimized Li@Al/Mg/V-based, Na@Mg/Y-based, and K@Al-based Hex-MBene (T = 300 K, time = 5 ps). Figure S9. The side views of Li_2_B_2_ (a) and hcp-Li bilayer (b). The variation of WFs with *D*_Li-Li_ in Li_2_B_2_ (c) and the variation of *E_ads_* of Li layer with WFs of Li_2_B_2_ (d) and *hcp*-Li bilayer (e). Figure S10. The ELFs of selected 12 *h*-MBenes. Figure S11. The ELFs of Li*_x_*M_2_B_2_ (M = Al, Mg, and V, *x* = 0, 2, 4, 6, and 8). Figure S12. The charge density difference of the first-layer adsorption of Li, Na, and K on Mg_2_B_2_, where blue regions indicate electron depletion and yellow-red regions represent electron accumulation.
