# Supplementary material for: Ultrahigh Storage Capacity of Alkali Metal Ions in Hexagonal Metal Borides with Orderly Multilayered Growth Mechanism
Source: Nanomaterials (Basel). 2025 Jun 8;15(12):886. doi: 10.3390/nano15120886 (PMC12195714; doi:10.3390/nano15120886)
Supplement: Supplementary file 1 [file nanomaterials-15-00886-s001.zip › nanomaterials-3673255-supplementary.pdf]

nanomaterials

MDPI

*Supporting Materials*

**Table S1.** The lattice constant, formation energy, elastic constants, Young's stiffness, and Poisson's ratio of selected 12 *h*-MBenes.

| Hex-MBene | Lattice constant (Å) | Formation energy (eV/atom) | Elastic constants (N/m) | Young's stiffness (N/m) | Poisson's ratio |
|---|---|---|---|---|---|
| $Li_2B_2$ | a =b=3.048 | -0.379 | $C_{11}$=$C_{22}$=145.2<br>$C_{12}$=$C_{21}$=-0.8<br>$C_{66}$=73.0 | 145.2 | -0.005 |
| $Mg_2B_2$ | a=b=3.091 | -0.109 | $C_{11}$=$C_{22}$=154.0<br>$C_{12}$=$C_{21}$=8.8<br>$C_{66}$=72.6 | 153.5 | 0.057 |
| $Al_2B_2$ | a=b=2.941 | 0.005 | $C_{11}$=$C_{22}$=222.8<br>$C_{12}$=$C_{21}$=35.7<br>$C_{66}$=93.6 | 217.1 | 0.160 |
| $Ca_2B_2$ | a=b=3.349 | -0.124 | $C_{11}$=$C_{22}$=113.8<br>$C_{12}$=$C_{21}$=16.8<br>$C_{66}$=48.5 | 111.3 | 0.148 |
| $Sc_2B_2$ | a=b=3.111 | -0.539 | $C_{11}$=$C_{22}$=207.8<br>$C_{12}$=$C_{21}$=18.3<br>$C_{66}$=94.8 | 206.2 | 0.088 |
| $Ti_2B_2$ | a=b=3.002 | -0.440 | $C_{11}$=$C_{22}$=240.9<br>$C_{12}$=$C_{21}$=15.2<br>$C_{66}$=73.0 | 239.9 | 0.063 |
| $V_2B_2$ | a=b=2.942 | -0.098 | $C_{11}$=$C_{22}$=220.1<br>$C_{12}$=$C_{21}$=46.7<br>$C_{66}$=86.7 | 210.2 | 0.212 |
| $Nb_2B_2$ | a=b=3.088 | -0.155 | $C_{11}$=$C_{22}$=209.5<br>$C_{12}$=$C_{21}$=82.4<br>$C_{66}$=63.6 | 177.1 | 0.393 |
| $Zr_2B_2$ | a=b=3.148 | -0.457 | $C_{11}$=$C_{22}$=227.8<br>$C_{12}$=$C_{21}$=47.5<br>$C_{66}$=90.1 | 217.9 | 0.209 |
| $Y_2B_2$ | a=b=3.271 | -0.386 | $C_{11}$=$C_{22}$=171.2<br>$C_{12}$=$C_{21}$=11.9<br>$C_{66}$=79.6 | 170.3 | 0.070 |
| $Hf_2B_2$ | a=b=3.105 | -0.430 | $C_{11}$=$C_{22}$=246.7<br>$C_{12}$=$C_{21}$=54.3<br>$C_{66}$=96.2 | 234.8 | 0.220 |
| $Ta_2B_2$ | a=b=3.062 | -0.070 | $C_{11}$=$C_{22}$=243.1<br>$C_{12}$=$C_{21}$=81.6<br>$C_{66}$=80.7 | 215.7 | 0.336 |

**Table S2.** The $E_{ads\text{-}step}$ of $Li_xM_2B_2$.

| $E_{step}$ **(eV, vs. Li)** | $x$**=0-2** | $x$**=2-4** | $x$**=4-6** | $x$**=6-8** | $x$**=8-10** |
|---|---|---|---|---|---|
| $Mg_2B_2$ | -0.317 | -0.015 | -0.014 | -0.005 | -0.006 |
| $Al_2B_2$ | -0.419 | -0.038 | -0.023 | -0.005 | -0.010 |

| | | | | | |
|---|---|---|---|---|---|
| $V_2B_2$ | -0.714 | -0.046 | -0.003 | -0.003 | -0.003 |
| $Sc_2B_2$ | -0.390 | -0.011 | -0.004 | 0.007 | |
| $Nb_2B_2$ | -0.625 | -0.048 | -0.007 | 0.003 | |
| $Ta_2B_2$ | -0.642 | -0.062 | -0.015 | 0.001 | |
| $Ti_2B_2$ | -0.651 | -0.016 | 0.004 | | |
| $Y_2B_2$ | -0.268 | -0.007 | 0.018 | | |
| $Zr_2B_2$ | -0.543 | -0.045 | 0.015 | | |
| $Hf_2B_2$ | -0.559 | -0.030 | 0.011 | | |
| $Ca_2B_2$ | -0.195 | 0.008 | | | |
| $Li_2B_2$ | 0.151 | | | | |

**Table S3.** The $E_{ads\text{-}step}$ of $Na_xM_2B_2$.

| $E_{step}$ **(eV, vs. Na)** | $x$**=0-1.5** | $x$**=1.5-3** | $x$**=3-4.5** | $x$**=4.5-6** |
|---|---|---|---|---|
| $Mg_2B_2$ | -0.327 | -0.027 | -0.002 | 0.003 |
| $Y_2B_2$ | -0.404 | -0.038 | -0.016 | 0.013 |
| $Hf_2B_2$ | -0.499 | -0.065 | -0.001 | 0.013 |
| $Al_2B_2$ | -0.247 | -0.005 | 0.020 | |
| $Sc_2B_2$ | -0.473 | -0.031 | 0.003 | |
| $Ti_2B_2$ | -0.633 | -0.105 | 0.007 | |
| $V_2B_2$ | -0.706 | -0.023 | 0.026 | |
| $Nb_2B_2$ | -0.575 | -0.083 | 0.004 | |
| $Zr_2B_2$ | -0.524 | -0.076 | 0.001 | |
| $Ta_2B_2$ | -0.573 | -0.063 | 0.002 | |
| $Ca_2B_2$ | -0.379 | 0.003 | | |
| $Li_2B_2$ | 0.243 | | | |

**Table S4.** The $E_{ads\text{-}step}$ of $K_xM_2B_2$.

| $E_{step}$ **(eV, vs. K)** | $x$**=0-0.667** | $x$**=0.667-1.333** | $x$**=1.333-2** |
|---|---|---|---|
| $Al_2B_2$ | -0.592 | -0.010 | 0.012 |
| $Mg_2B_2$ | -0.558 | 0.017 | |
| $Ca_2B_2$ | -0.514 | 0.146 | |
| $Sc_2B_2$ | -0.718 | 0.080 | |
| $Ti_2B_2$ | -1.022 | 0.036 | |
| $V_2B_2$ | -1.157 | 0.047 | |
| $Nb_2B_2$ | -0.964 | 0.078 | |
| $Zr_2B_2$ | -0.897 | 0.076 | |
| $Y_2B_2$ | -0.611 | 0.137 | |
| $Hf_2B_2$ | -0.894 | 0.052 | |
| $Ta_2B_2$ | -0.995 | 0.058 | |
| $Li_2B_2$ | -0.138 | 0.050 | |

**Table S5.** The lattice mismatch rate of $Li_xM_2B_2$.

| **Lattice mismatch rate (%)** | **$M_2B_2$** | **$Li_2M_2B_2$** | **$Li_4M_2B_2$** | **$Li_6M_2B_2$** | **$Li_8M_2B_2$** | **$Li_{10}M_2B_2$** |
|---|---|---|---|---|---|---|
| $Mg_2B_2$ | 3.06 | 3.32 | 2.74 | 2.67 | 2.39 | 2.15 |
| $Al_2B_2$ | -1.92 | -0.31 | -0.59 | -0.51 | -0.51 | -0.58 |
| $V_2B_2$ | -1.91 | -1.02 | -1.09 | -1.24 | -1.25 | -1.22 |
| $Sc_2B_2$ | 3.75 | 4.61 | 4.11 | 3.75 | | |
| $Nb_2B_2$ | 2.95 | 3.35 | 3.57 | 3.10 | | |
| $Ta_2B_2$ | 2.11 | 1.47 | 2.99 | 2.58 | | |
| $Ti_2B_2$ | 0.12 | 0.19 | 0.16 | | | |
| $Zr_2B_2$ | 4.97 | 5.82 | 5.30 | | | |
| $Y_2B_2$ | 9.07 | 9.53 | 8.71 | | | |
| $Hf_2B_2$ | 3.53 | 4.51 | 4.20 | | | |
| $Ca_2B_2$ | 11.64 | 10.45 | | | | |
| $Li_2B_2$ | 1.62 | | | | | |

**Table S6.** The lattice mismatch rate of $Na_xM_2B_2$.

| Lattice mismatch rate (%) | $M_2B_2$ | $Na_{1.5}M_2B_2$ | $Na_3M_2B_2$ | $Na_{4.5}M_2B_2$ |
|---|---|---|---|---|
| $Mg_2B_2$ | -4.59 | -4.14 | -3.95 | -3.78 |
| $Y_2B_2$ | 0.97 | 1.42 | 1.16 | 0.93 |
| $Hf_2B_2$ | -4.16 | -3.51 | -3.25 | -3.21 |
| $Al_2B_2$ | -9.21 | -8.18 | -7.65 | |
| $Sc_2B_2$ | -3.95 | -3.23 | -3.22 | |
| $Ti_2B_2$ | -7.31 | -7.26 | -7.06 | |
| $V_2B_2$ | -9.19 | -8.22 | -7.97 | |
| $Nb_2B_2$ | -4.69 | -3.58 | -3.62 | |
| $Zr_2B_2$ | -2.82 | -2.15 | -2.11 | |
| $Ta_2B_2$ | -5.47 | -4.67 | -4.17 | |
| $Ca_2B_2$ | 3.35 | 2.67 | | |
| $Li_2B_2$ | 5.92 | | | |

**Table S7.** The lattice mismatch rate of $K_xM_2B_2$.

| Lattice mismatch rate (%) | $M_2B_2$ | $K_{0.67}M_2B_2$ | $K_{1.33}M_2B_2$ |
|---|---|---|---|
| $Al_2B_2$ | 8.75 | 9.19 | 8.99 |
| $Mg_2B_2$ | 14.14 | 14.06 | |
| $Ca_2B_2$ | 24.04 | 22.92 | |
| $Sc_2B_2$ | 15.19 | 15.30 | |
| $Ti_2B_2$ | 11.13 | 10.57 | |
| $V_2B_2$ | 8.88 | 9.11 | |
| $Nb_2B_2$ | 14.25 | 14.71 | |
| $Zr_2B_2$ | 16.56 | 16.81 | |
| $Y_2B_2$ | 21.13 | 21.11 | |
| $Hf_2B_2$ | 14.91 | 15.24 | |
| $Ta_2B_2$ | 13.42 | 14.01 | |
| $Li_2B_2$ | 12.76 | 11.72 | |

**Table S8.** The work function of $Li_xM_2B_2$.

| Work function (eV) | $M_2B_2$ | $Li_2M_2B_2$ | $Li_4M_2B_2$ | $Li_6M_2B_2$ | $Li_8M_2B_2$ | $Li_{10}M_2B_2$ |
|---|---|---|---|---|---|---|
| $Mg_2B_2$ | 4.12 | 3.46 | 3.27 | 3.10 | 3.14 | 3.22 |
| $Al_2B_2$ | 4.39 | 3.77 | 3.51 | 3.36 | 3.33 | 3.33 |
| $V_2B_2$ | 4.67 | 3.18 | 3.10 | 3.25 | 3.31 | 3.34 |
| $Sc_2B_2$ | 3.89 | 3.04 | 3.08 | 3.18 | | |
| $Nb_2B_2$ | 4.53 | 3.05 | 3.05 | 3.18 | | |
| $Ta_2B_2$ | 4.79 | 3.56 | 3.01 | 3.15 | | |
| $Ti_2B_2$ | 4.09 | 2.89 | 2.99 | | | |
| $Zr_2B_2$ | 4.59 | 2.76 | 2.98 | | | |
| $Y_2B_2$ | 3.76 | 2.87 | 3.00 | | | |
| $Hf_2B_2$ | 4.74 | 2.91 | 2.92 | | | |
| $Ca_2B_2$ | 3.41 | 3.11 | | | | |
| $Li_2B_2$ | 2.51 | | | | | |

**Table S9.** The work function of $Na_xM_2B_2$.

| Work function (eV) | $M_2B_2$ | $Na_{1.5}M_2B_2$ | $Na_3M_2B_2$ | $Na_{4.5}M_2B_2$ |
|---|---|---|---|---|
| $Mg_2B_2$ | 4.12 | 3.14 | 2.96 | 2.89 |
| $Y_2B_2$ | 3.76 | 2.47 | 2.65 | 2.83 |
| $Hf_2B_2$ | 4.74 | 3.02 | 2.49 | 2.73 |
| $Al_2B_2$ | 4.39 | 3.34 | 3.12 | |
| $Sc_2B_2$ | 3.89 | 2.72 | 2.67 | |
| $Ti_2B_2$ | 4.09 | 2.89 | 2.70 | |
| $V_2B_2$ | 4.67 | 2.90 | 2.73 | |
| $Nb_2B_2$ | 4.53 | 2.70 | 2.63 | |
| $Zr_2B_2$ | 4.59 | 2.83 | 2.49 | |
| $Ta_2B_2$ | 4.79 | 3.19 | 2.66 | |
| $Ca_2B_2$ | 3.41 | 2.58 | | |
| $Li_2B_2$ | 2.51 | | | |

**Table S10.** The work function of $K_xM_2B_2$.

| Work function (eV) | $M_2B_2$ | $K_{0.67}M_2B_2$ | $K_{1.33}M_2B_2$ |
|---|---|---|---|
| $Al_2B_2$ | 4.39 | 2.46 | 2.380 |
| $Mg_2B_2$ | 4.12 | 2.490 | |
| $Ca_2B_2$ | 3.41 | 2.470 | |
| $Sc_2B_2$ | 3.89 | 2.500 | |
| $Ti_2B_2$ | 4.09 | 2.64 | |
| $V_2B_2$ | 4.67 | 2.65 | |
| $Nb_2B_2$ | 4.53 | 2.64 | |
| $Zr_2B_2$ | 4.59 | 2.59 | |
| $Y_2B_2$ | 3.76 | 2.39 | |
| $Hf_2B_2$ | 4.74 | 2.67 | |
| $Ta_2B_2$ | 4.79 | 2.74 | |
| $Li_2B_2$ | 2.51 | 2.01 | |

**Table S11.** The Bader charge analysis ($\Delta e$) per alkali metal atom in the first adsorbed alkali metal layer, where a negative value indicates electron loss.

| AM, $\Delta e$ | Li | Na | K |
|---|---|---|---|
| $Mg_2B_2$ | -0.77 | -0.39 | -0.51 |
| $Al_2B_2$ | -0.75 | -0.39 | -0.50 |
| $V_2B_2$ | -0.77 | -0.48 | -0.56 |
| $Sc_2B_2$ | -0.78 | -0.45 | -0.52 |
| $Nb_2B_2$ | -0.78 | -0.51 | -0.58 |
| $Ta_2B_2$ | -0.77 | -0.48 | -0.60 |
| $Ti_2B_2$ | -0.78 | -0.48 | -0.56 |
| $Y_2B_2$ | -0.76 | -0.44 | -0.51 |
| $Zr_2B_2$ | -0.79 | -0.49 | -0.58 |
| $Hf_2B_2$ | -0.78 | -0.48 | -0.58 |
| $Ca_2B_2$ | -0.75 | -0.37 | -0.48 |
| $Li_2B_2$ | - | - | -0.26 |

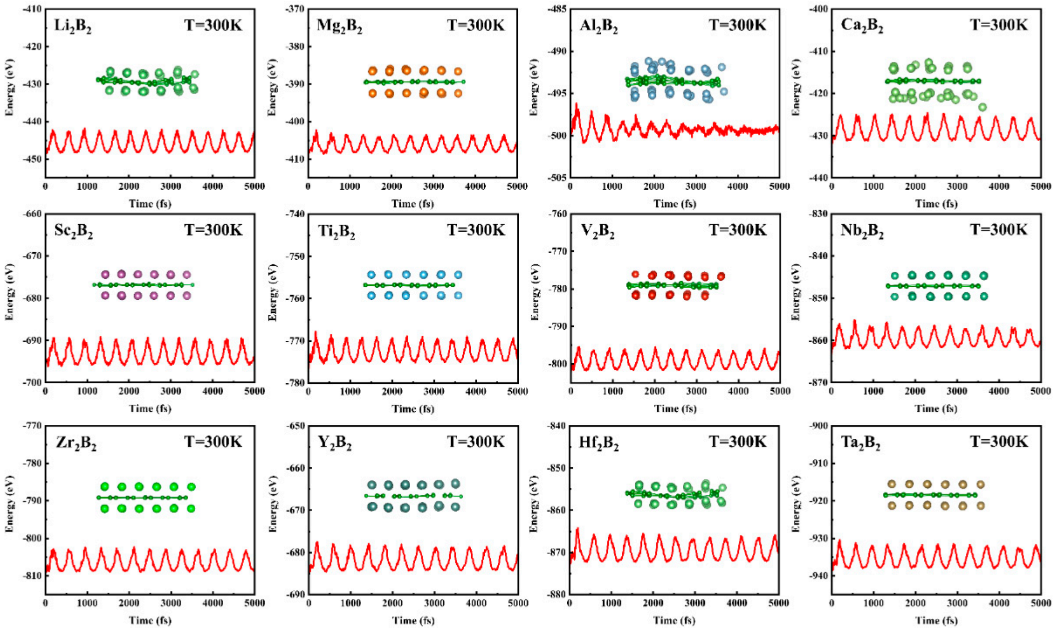


**Figure S1.** The AIMD simulations of selected 12 *h*-MBenes (T = 300 K, time = 5 ps).

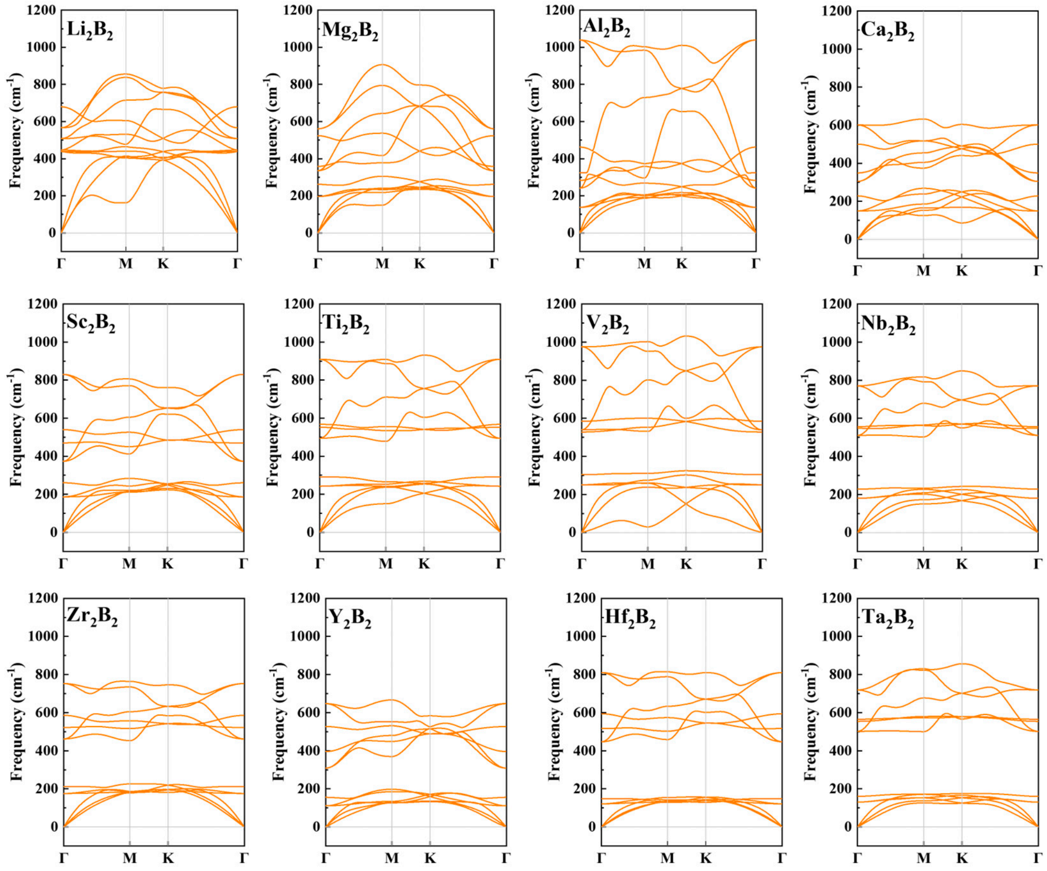


**Figure S2.** The phonon spectra of selected 12 *h*-MBenes.

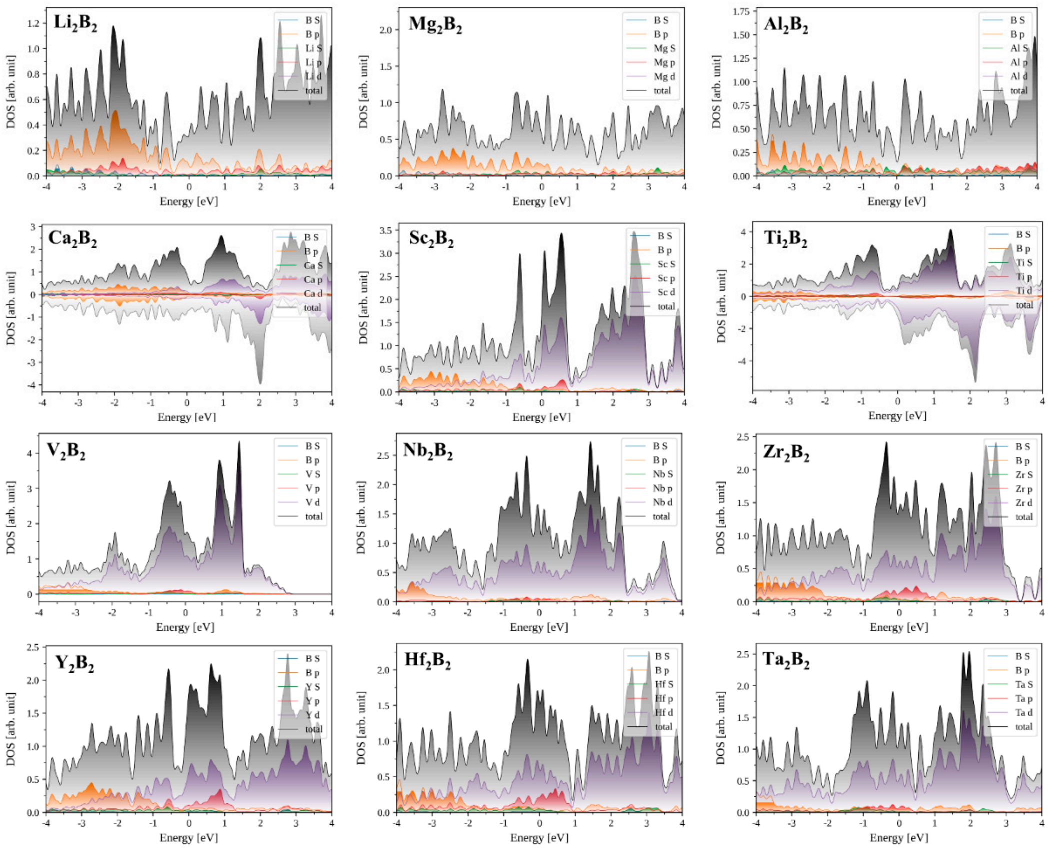


**Figure S3.** The PDOS of selected 12 *h*-MBenes under the PBE level.

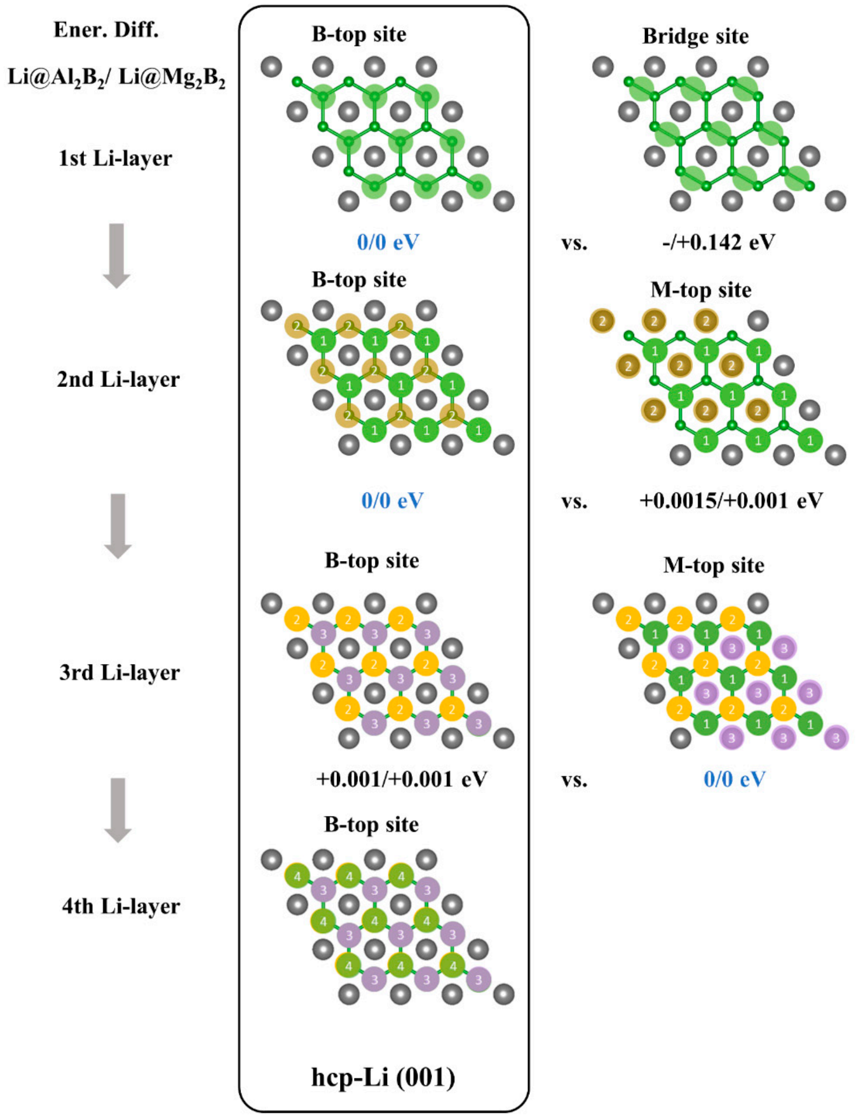


**Figure S4.** The adsorption configurations and energy differences of each Li layer on $Al_2B_2$ and $Mg_2B_2$ substrates, with the most stable structure in each model as the energy reference.

The different stacking modes of Li during the layer-by-layer adsorption process on the surfaces of $Mg_2B_2$ and $Al_2B_2$ are investigated. As shown in Figure S4, for the adsorption of the first Li layer, the B-top site exhibits the lowest energy (for $Al_2B_2$, Li is initially placed at the bridge site and moves to the B-top position after relaxation). From the second to the third adsorption layers, as long as the newly adsorbed Li forms a three-coordinated configuration with the pre-adsorbed Li, the energy differences between different stacking modes become negligible (<1.5 meV). Therefore, we chose to stack the alkali metal layer by layer on the $M_2B_2$ surface following the (001) plane of the hcp phase.

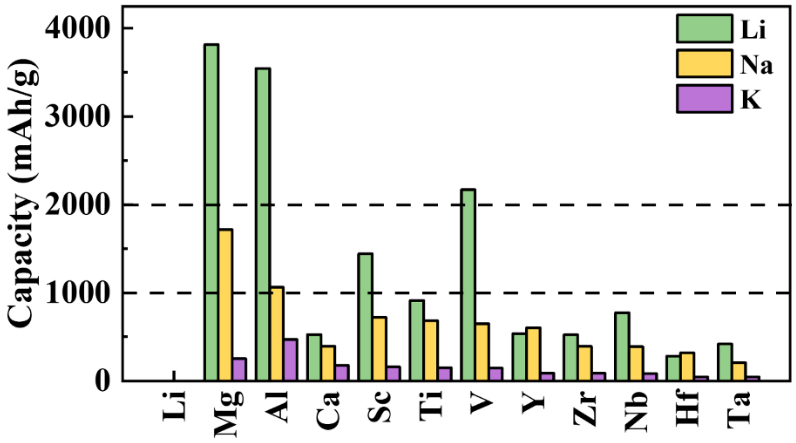


**Figure S5.** Theoretical specific capacity (mAh·g$^{-1}$) comparison of various *h*-MBenes for alkali metals (Li/Na/K).

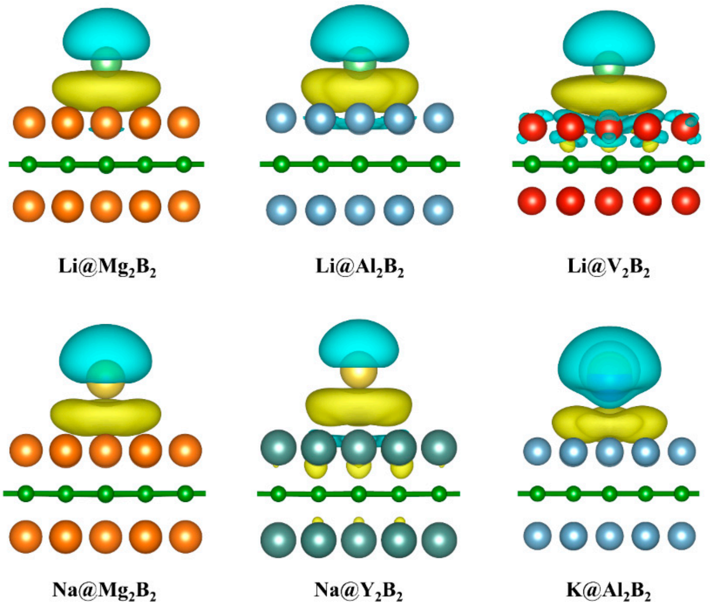


**Figure S6.** Charge density difference of various alkali metal ions (Li/Na/K) adsorbed on different *h*-MBene surfaces.

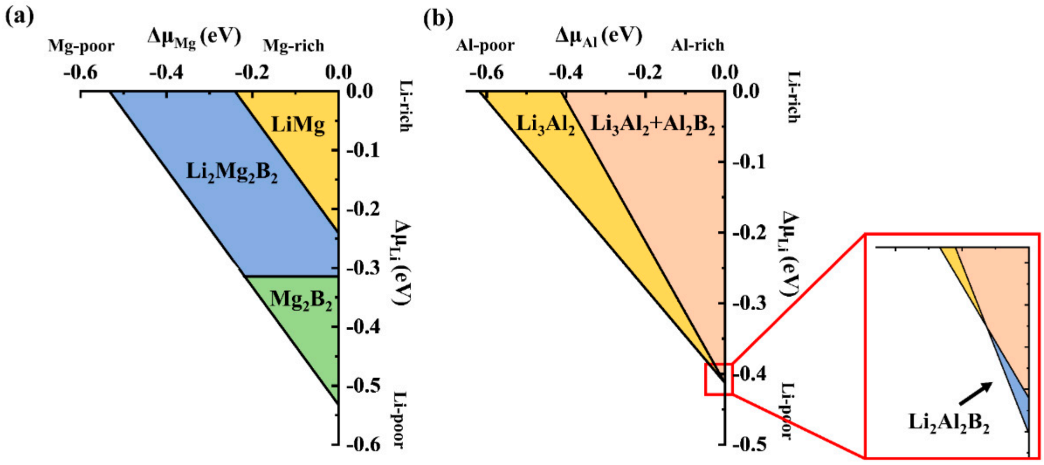

**Figure S7.** Thermodynamic phase diagrams of lithiated (a) $Li_2Mg_2B_2$ and (b) $Li_2Al_2B_2$ structures.

## Thermodynamic diagram

We assumed that $Li_2M_2B_2$ (M = Mg/Al) can be formed during the lithiation process, while the chemical potentials of Li, M, and B required following the thermodynamic equilibrium as follows:

$$\Delta H(Li_2M_2B_2) = E_{total} - 2\mu_{Li} - 2\mu_M - 2\mu_B \tag{1}$$

where $E_{total}$, $\mu_{Li}$, $\mu_M$, and $\mu_B$ are the total energy of $Li_2M_2B_2$ and the energies of Li, Mg, and Al in metal bulk and B in the $\beta_{12}$ borophene monolayer.

$$2\Delta\mu_{Li} + 2\Delta\mu_M + 2\Delta\mu_B = \Delta H(Li_2M_2B_2) \tag{2}$$

where $\Delta H(Li_2M_2B_2)$ is the formation enthalpy of $Li_2M_2B_2$ (M = Mg/Al). To avoid the formation of Li-Al and Li-Mg alloys as well as the formation of M, Ca, and P crystals, the chemical potentials also satisfy the following constraints:

$$3\Delta\mu_{Li} + 2\Delta\mu_{Al} \leq \Delta H(Li_3Al_2) \tag{3}$$

$$\Delta\mu_{Li} + \Delta\mu_{Mg} \leq \Delta H(LiMg) \tag{4}$$

$$2\Delta\mu_M + 2\Delta\mu_B \leq \Delta H(M_2B_2) \tag{5}$$

$$\Delta\mu_M \leq 0, \qquad \Delta\mu_{Li} \leq 0, \qquad \Delta\mu_B \leq 0 \tag{6}$$

where $\Delta H(Li_3Al_2)$ and $\Delta H(LiMg)$ are the formation enthalpies of $Li_3Al_2$ and LiMg, respectively. Using Eq. (2)–(6), the thermodynamic phase diagrams of lithiated $Li_2Mg_2B_2$ and $Li_2Al_2B_2$ can be obtained.

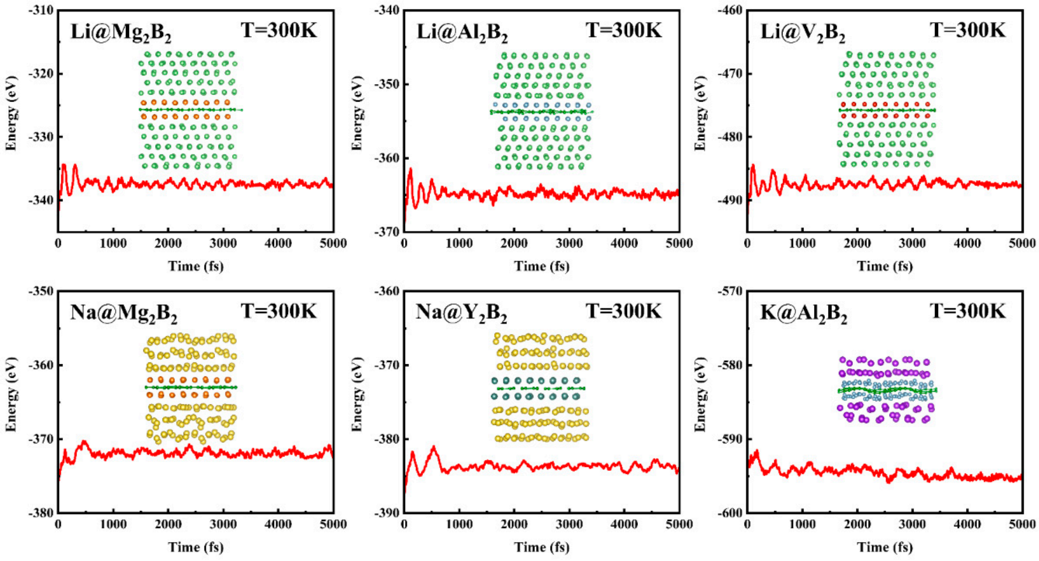


**Figure S8.** AIMD simulations of the saturated adsorption systems for performance-optimized Li@Al/Mg/V-based, Na@Mg/Y-based, and K@Al-based Hex-MBenes (T = 300 K, time = 5 ps).

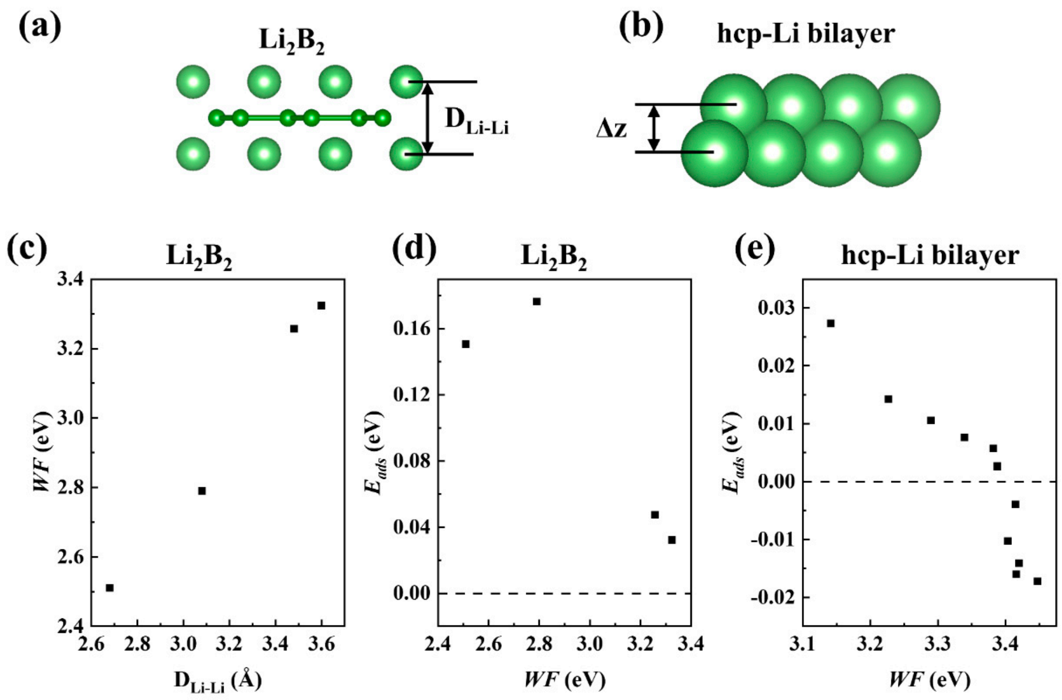


**Figure S9.** Side views of $Li_2B_2$ (a) and hcp-Li (b) bilayers. The variation in WFs with $D_{Li-Li}$ in $Li_2B_2$ (c) and the variation in $E_{ads}$ of the Li layer with WFs of $Li_2B_2$ (d) and hcp-Li (e) bilayers.

The work function (WF) of the $Li_2B_2$ and hcp-Li bilayers can be artificially modulated by adjusting the interlayer spacing between Li layers in $Li_2B_2$ and hcp-Li bilayers (Figure S7a and S7b). Adsorption tests of the first Li layer of $Li_2B_2$ and hcp-Li bilayers reveal that the adsorption energy is positive when the WF is below the hcp-Li bulk reference value ($WF_{Li}$). As the WF increases and approaches $WF_{Li}$, the adsorption energy decreases correspondingly. When the WF exceeds $WF_{Li}$, the adsorption energy becomes negative.

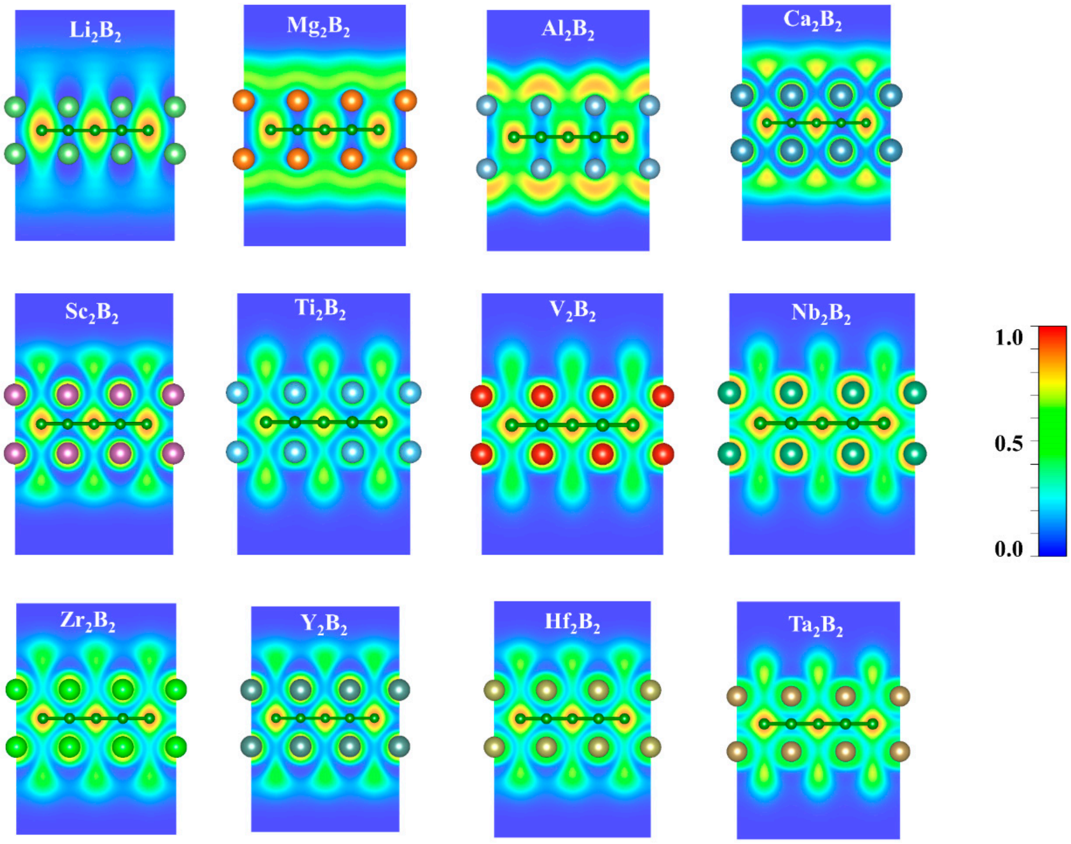


**Figure S10.** The ELFs of selected 12 *h*-MBenes.

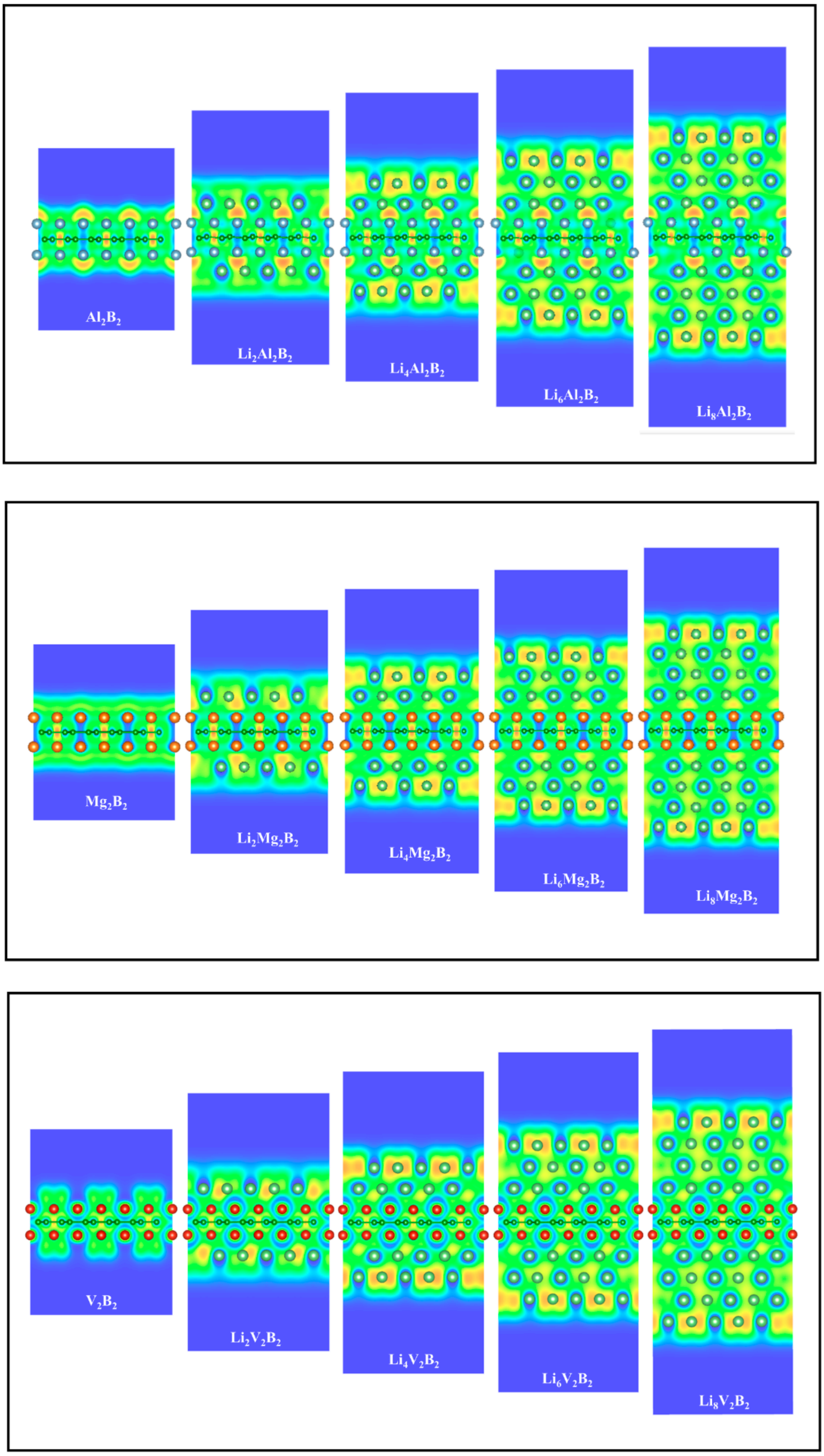


**Figure S11.** The ELFs of $Li_xM_2B_2$ (M = Al, Mg, and V, x = 0, 2, 4, 6, and 8).

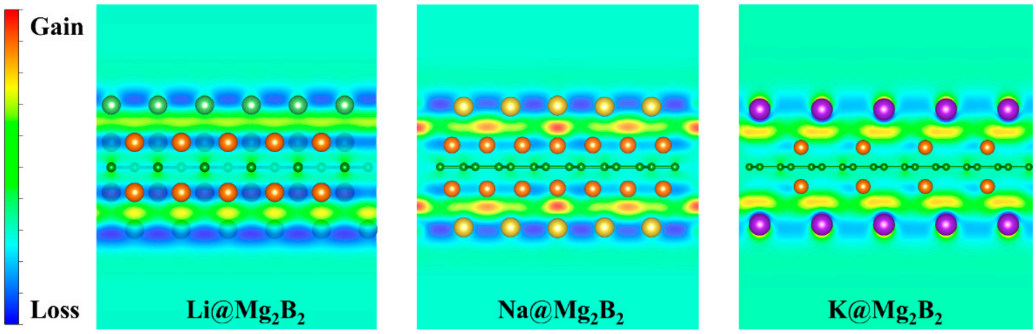


**Figure S12.** The charge density difference of the first-layer adsorption of Li, Na, and K on $Mg_2B_2$, where blue regions indicate electron depletion and yellow–red regions represent electron accumulation.
